# Internal fixation versus hip arthroplasty in patients with nondisplaced femoral neck fractures: short-term results from a geriatric trauma registry

**DOI:** 10.1007/s00068-021-01801-1

**Published:** 2021-10-05

**Authors:** Markus Laubach, Felix M. Bläsius, Ruth Volland, Matthias Knobe, Christian D. Weber, Frank Hildebrand, Miguel Pishnamaz, Registry for Geriatric Trauma DGU, Matthias Knobe

**Affiliations:** 1 Department of Orthopaedics, Trauma and Reconstructive Surgery, RWTH Aachen University Hospital, Pauwelsstraße 30, 52074 Aachen, Germany; 2 School of Mechanical, Medical and Process Engineering, Faculty of Engineering, Queensland University of Technology, Brisbane, 4059 Australia; 3 AUC-Academy for Trauma Surgery (AUC), Munich, Germany; 4 Department of Orthopedic and Trauma Surgery, Lucerne Cantonal Hospital, Lucerne, Switzerland; 5 Working Committee on Geriatric Trauma Registry (AK ATR) of the German Trauma Society (DGU), Berlin, Germany

**Keywords:** Health-related quality of life, Hip fracture, Treatment, Elderly, Reoperation, Mobility

## Abstract

**Purpose:**

To determine whether internal fixation (IF) or hip arthroplasty (HA) is associated with superior outcomes in geriatric nondisplaced femoral neck fracture (FNF) patients.

**Methods:**

Data from the Registry for Geriatric Trauma of the German Trauma Society (ATR-DGU) were analyzed (IF Group 449 and HA Group 1278 patients). In-hospital care and a 120-day postoperative follow-up were conducted. Primary outcomes, including mobility, residential status, reoperation rate, and a generic health status measure (EQ-5D score), and the secondary outcome of mortality were compared between groups. Multivariable analyses were performed to assess independent treatment group associations (odds ratios, ORs) with the primary and secondary end points.

**Results:**

Patients in the HA group were older (83 vs. 81 years, *p* < 0.001) and scored higher on the Identification of Seniors at Risk screening (3 vs. 2, *p* < 0.001). We observed no differences in residential status, reoperation rate, EQ-5D score, or mortality between groups. After adjusting for key covariates, including prefracture ambulatory capacity, the mobility of patients in the HA group was more frequently impaired at the 120-day follow-up (OR 2.28, 95% confidence interval = 1.11–4.74).

**Conclusion:**

Treatment with HA compared to treatment with IF led to a more than twofold increase in the adjusted odds of impaired ambulation at the short-term follow-up, while no significant associations with residential status, reoperation rate, EQ-5D index score, or mortality were observed. Thus, IF for geriatric nondisplaced FNFs was associated with superior mobility 120 days after surgery. However, before definitive treatment recommendations can be made, prospective, randomized, long-term studies must be performed to confirm our findings.

**Supplementary Information:**

The online version contains supplementary material available at 10.1007/s00068-021-01801-1.

## Introduction

Femoral neck fractures (FNFs) are one of the most common injuries (> 50% of all hip fractures) among geriatric trauma patients and are associated with a significant health care burden as well as detrimental effects on quality of life [1–3]. A threefold functional decline and increases in mortality during the first year ranging from 8.4% to 36.0% have been observed in hip fracture patients when compared to a prospectively studied population of uninjured individuals [4, 5].

Joint replacement techniques, such as total hip replacement (THR) or hemiarthroplasty, are well established as treatments for displaced FNFs [6–8]. For the treatment of nondisplaced FNFs, however, no therapy recommendations based on high-class evidence have been established. In a retrospective study assessing the Norwegian hip fracture register, no clinically relevant differences between screw osteosynthesis for nondisplaced FNFs and hemiarthroplasty for displaced FNFs were observed [9]. Notably, fewer wound infections, less blood loss, and shorter surgery times have been described for the treatment of FNFs with internal fixation (IF) than for hip arthroplasty (HA) [10]. In addition, the risk of nonunion and avascular necrosis of the femoral head (AVN) after osteosynthetic treatment of nondisplaced FNFs has been considered low in the past [11]. However, these findings have been challenged by prospective trials, some of which have yielded beneficial and others unfavorable clinical outcomes of hemiarthroplasty when compared to IF among patients with nondisplaced FNFs [12–14]. Furthermore, the literature indicates an overall risk of reoperation of 14.1% and conversion to HA rates of up to 16% among elderly nondisplaced FNF patients treated with IF [15, 16]. Notably, the health care costs associated with conversion from IF to HA are significantly higher than those for primary joint replacement [17].

Thus, the controversy surrounding the best treatment of nondisplaced FNF has not yet been resolved. The present study aimed to compare mobility, residential status, reoperation rate, and health-related quality of life (HRQoL) as well as early mortality associated with IF and HA for nondisplaced FNFs by evaluating data from a geriatric trauma registry.

## Methods

### Study design and patient selection

**Fig. 1:**
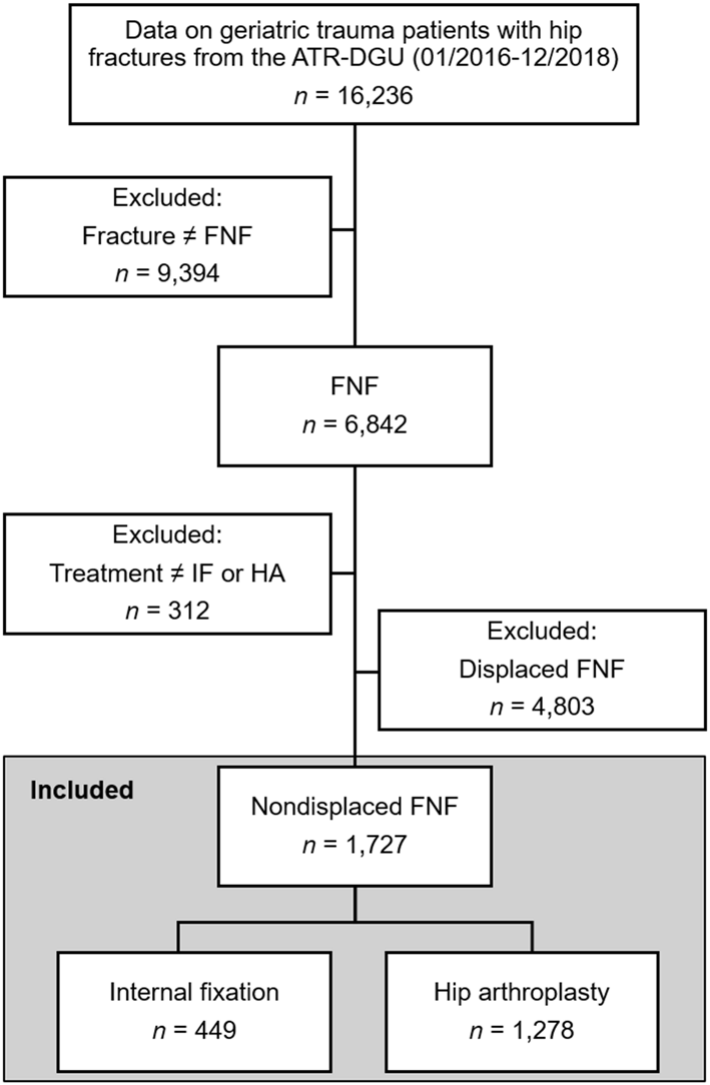
Study design and patient selection flow chart. ATR-DGU, Registry for Geriatric Trauma of the German Trauma Society; *FNF* femoral neck fracture, *HA* hip arthroplasty, *IF* internal fixation

We performed a retrospective register-based observational study with datasets from the Registry for Geriatric Trauma of the German Trauma Society (ATR-DGU) including geriatric trauma patients with hip fractures admitted to 74 hospitals between 1 January 2016 and 31 December 2018 (*n* = 16,236). After the exclusion of all non-FNF patients (*n* = 9394), displaced FNF patients (*n* = 4803) and patients who had undergone treatments other than IF or HA (*n* = 312), the final cohort of nondisplaced FNF patients (Garden Types I and II fractures; *n* = 1727) included only patients treated with either IF (*n *= 449) or HA (*n* = 1278, Fig. 1). Data from the ATR-DGU comprise pseudonymized and standardized documentation of geriatric patients (≥ 70 years of age) with surgically treated hip fractures collected at five consecutive time points: upon hospital admission, preoperatively, intraoperatively, during the first postoperative week, and at discharge/transfer. Furthermore, on postoperative Day 120, an additional optional follow-up was conducted. Upon hospital admission, the patients were assessed once with the modified and validated Identification of Seniors at Risk (ISAR) screening tool [18, 19]. The ISAR score indicates the risk of adverse health outcomes, including mortality, functional decline, readmission, and institutionalization, and ranges from 0 (low risk) to 6 (high risk) points. The total number of patients with prefracture anticoagulation was recorded, and consequently, in the case of intake of anticoagulants, the individual anticoagulant medication was reported. Briefly, antiplatelet drugs (APDs) include acetylsalicylic acid and P2Y_12_ receptor blockers, vitamin K antagonists (VKAs) include phenprocoumon and warfarin, and direct oral anticoagulants (DOACs) include factor IIa inhibitors and factor Xa inhibitors. All patients provided written informed consent for participation in the registry (ATR-DGU) after receiving spoken and written information. The present study complies with the RECORD statement [20] and is in line with the publication guidelines of the ATR-DGU [21] registered under project ID 2019–008.

### Primary and secondary outcomes

Primary outcomes included mobility and residential status, reoperation rate, and HRQoL. The prefracture mobility status was categorized as ambulation without impairment or impaired ambulation depending on whether assistive devices were needed to support walking. The ambulation without impairment category included patients with the ability to walk without assistive devices. The category of impaired ambulation included patients who used walking sticks, crutches, a walker or any other mobility device either inside or outside the residency or who had no functional walking ability (i.e., no possible use of lower extremities). Furthermore, the residential status of the patients was documented and categorized as not institutionalized (community-dwelling) or institutionalized (residents of nursing homes and hospitals) for further analysis. A reoperation was defined as any surgical procedure performed due to complications after the primary (index) surgery—either during in-hospital care or during the first 120 days after the index surgery. HRQoL was measured by the non-disease-specific European Quality of Life-5 Dimensions (EQ-5D) instrument [22]. The EuroQol Group developed the standardized EQ-5D questionnaire, which expresses each respondent’s health status through a one-dimensional measure ranging from 0 (very poor) to 1 (the best possible health) [23]. The survey datasets available in this study were based on the original three-level version of the EQ-5D questionnaire (EQ-5D-3L), hereafter referred to as the EQ-5D questionnaire. Responses to the five questions of the EQ-5D questionnaire were transformed into a single HRQoL value using the time trade-off algorithm, which was validated for use in Germany [24]. The baseline EQ-5D questionnaire was administered during the first week of hospital admission and repeated prospectively during a routine follow-up of 120 days after surgery either in the outpatient clinic or via telephone interview. Mortality, both during in-hospital care and 120 days after surgery, was the secondary outcome.

### Statistical analyses

The baseline characteristics and outcome variables of the study population are provided using descriptive statistics. The data are presented as medians with interquartile ranges (IQRs) for continuous variables or percentages (%) for categorical variables. A Pearson chi-square test was used to compare categorical variables with more than five expected observations, and Fisher’s exact test was applied for categorical variables with fewer than five expected observations. A Mann–Whitney *U* test served to compare continuous variables. While adjusting for the key covariates of sex, age, prefracture residential and mobility status, ISAR scores, intake of prefracture anticoagulation, and ASA class (American Society of Anesthesiologists classification), multivariable logistic or linear regression analyses were applied to assess the associations of surgical treatment with in-hospital mortality and EQ-5D index scores as well as mortality, reoperation rate, residential and mobility status, and EQ-5D index scores 120 days after surgery. Odds ratios (ORs) or regression coefficients (ẞ) are presented with their respective 95% confidence intervals (CIs). Two-tailed *p* values < 0.05 were considered significant. All analyses were performed using R statistical software (version 4.0.2; R Foundation for Statistical Computing, Vienna, Austria).

## Results

The type of osteosynthesis (IF) and endoprosthesis (HA) and patients’ demographics as well as baseline characteristics are summarized in Table 1. Compared to patients in the IF group (*n* = 449, 66.0% female), patients in the HA group (*n* = 1278, 68.2% female) had a higher median age (83 years vs. 81 years, *p* < 0.001) and higher median ISAR scores (3 vs. 2, *p* < 0.001) and were less frequently able to ambulate without impairment prior to FNF (36.0% vs. 43.0% *p* = 0.012). Sex and residential status prior to fracture were not significantly different between groups. In the IF group, patients were prescribed APDs more frequently (79% vs. 65%), while in contrast, fewer VKAs (11% vs. 17%) and fewer DOACs (10% vs. 17%) were taken (*p* = 0.002). Patients with impaired mobility before their hip fractures consisted mainly of those using crutches for ambulation in both the IF (24.1%) and HA (28.9%) groups. Before sustaining FNFs, 3.5% of patients in the IF group and 3.7% in the HA group were unable to ambulate. Both the median time from hospital admission to surgery and the duration of in-hospital care (length of stay, LOS) were shorter in the IF group (16.4 h vs. 21.4 h, *p* < 0.001 and 14.0 days vs. 15.1 days, *p* < 0.001, respectively).

**Table 1:** Patient demographics and baseline characteristics^a^

Baseline characteristics	Internal fixation (*n* = 449)	Hip arthroplasty (*n* = 1278)	*p *value
Age (years), median (IQR)^d^	81 (76–87)	83 (79–88)	< 0.001^b^
Female, no./total no. (%)	304/446 (68.2%)	839/1272 (66.0%)	0.430
ISAR score, median (IQR)^e^	2 (1–3)	3 (2–4)	< 0.001^b^
Prefracture anticoagulation, no./total no. (%)	212/430 (49.3%)	619/1200 (51.6%)	0.450
Anticoagulant medication, no./total no. (%)			0.002
Antiplatelet drugs (APD)	166/212 (79%)	394/619 (65%)	
Vitamin K antagonists (VKA)	23/212 (11%)	103/619 (17%)	
Direct oral anticoagulants (DOAC)	22/212 (10%)	105/619 (17%)	
Other	1/212 (1%)	17/619 (1%)	
ASA class, no./total no. (%)			0.334^c^
ASA 1 healthy	15/448 (3.4%)	34/1264 (2.7%)	
ASA 2 mild, systemic disease	114/448 (25.5%)	323/1264 (25.6%)	
ASA 3 severe, systemic disease	292/448 (65.2%)	820/1264 (64.9%)	
ASA 4 incapacitating disease	27/448 (6.0%)	96/1264 (7.6%)	
ASA 5 moribund	0/448	1/1264 (0.1%)	
Procedure type			
Multiple cancellous screws	41.4%		
Single large-diameter screw (dynamic hip screw) with side-plate	33.4%		
Nail fixation	25.2%		
Total hip replacement		14.3%	
Hemiarthroplasty		85.7%	

*ASA class* American Society of Anesthesiologists classification, *IQR* interquartile range, *ISAR* Identification of Seniors at Risk

^a^Pearson’s chi-squared test unless otherwise specified

^b^Mann–Whitney *U* test

^c^Fisher’s exact test

^d^Data missing for seven patients in the internal fixation group and 13 patients in the hip arthroplasty group

^e^Data missing for 128 patients in the internal fixation group and 427 patients in the hip arthroplasty group

### Primary and secondary outcomes

Patients who received IF were more frequently able to ambulate without any impairment at 120 days after surgery than patients in the HA group (19.6% vs. 10.5%, *p* = 0.003). The number of institutionalized patients at follow-up was lower in the IF group (29.3% vs. 38.2%, *p* = 0.039). We found no difference in terms of reoperation rates during in-hospital care (IF 2.5%, HA 2.6%, *p* = 1.000) or during the 120-day follow-up period (IF 5.9%, HA 4.0%, *p* = 0.310). Notably, however, in the IF group, requiring secondary THR or hemiarthroplasty was the major cause for reoperation during in-hospital care (3 out of 12 events) and during 120 days of follow-up (9 out of 17 events). The types of reoperation are depicted in Supplement 1 of the supplementary material. The median EQ-5D index score on day seven after surgery was 0.70 in both groups (IF 0.29–0.79, HA 0.29–0.70, *p* = 0.769). At the 120-day follow-up, the EQ-5D index score of the IF group was 0.81 (0.5–1.0) and that of the HA group was 0.80 (0.5–0.9, *p* = 0.095). Mortality rates during in-hospital care (IF 3.6%, HA 3.7%, *p* = 1.000) and during the 120-day follow-up period were similar between the groups (IF 7.5%, HA 9.7%, *p* = 0.468). The primary and secondary outcomes are summarized in Table 2.

**Table 2:** Primary and secondary outcomes^a^

Outcome measure/time points	Internal fixation (*n* = 449)	Hip arthroplasty (*n* = 1278)	*p *value
Ambulation without impairment, no./total no. (%)			
Prefracture	182/423 (43.0%)	410/1193 (34.4%)	0.012
120 days after surgery	38/194 (19.6%)	47/447 (10.5%)	0.003
Institutionalized, no./total no. (%)			
Prefracture	106/440 (24.1%)	320/1242 (25.8%)	0.524
120 days after surgery	54/184 (29.3%)	146/382 (38.2%)	0.039
Reoperation, no./total no. (%)			
In-hospital care	11/447 (2.5%)	33/1275 (2.6%)	1.000 ^b^
120 days after surgery	14/239 (5.9%)	24/606 (4.0%)	0.310^b^
EQ-5D index score, median (IQR)			
In-hospital care^d^	0.70 (0.29–0.79)	0.70 (0.29–0.70)	0.769^c^
120 days after surgery^e^	0.81 (0.5–0.9)	0.80 (0.5–0.9)	0.095^c^
Mortality, no./total no. (%)			
In-hospital care	16/439 (3.6%)	46/1245 (3.7%)	1.000
120 days after surgery	15/199 (7.5%)	41/423 (9.7%)	0.468

*EQ-5D* European Quality of Life-5 Dimensions, *IQR* interquartile range

^a^Fisher’s exact test unless otherwise specified

^b^Pearson’s chi-squared test

^c^Mann–Whitney *U* test

^d^Data missing for 104 patients in the internal fixation group and 318 patients in the hip arthroplasty group

^e^Data missing for 292 patients in the internal fixation group and 957 patients in the hip arthroplasty group

### Multivariable regression analyses of surgical treatment as a predictor of primary and secondary outcomes

**Fig. 2:**
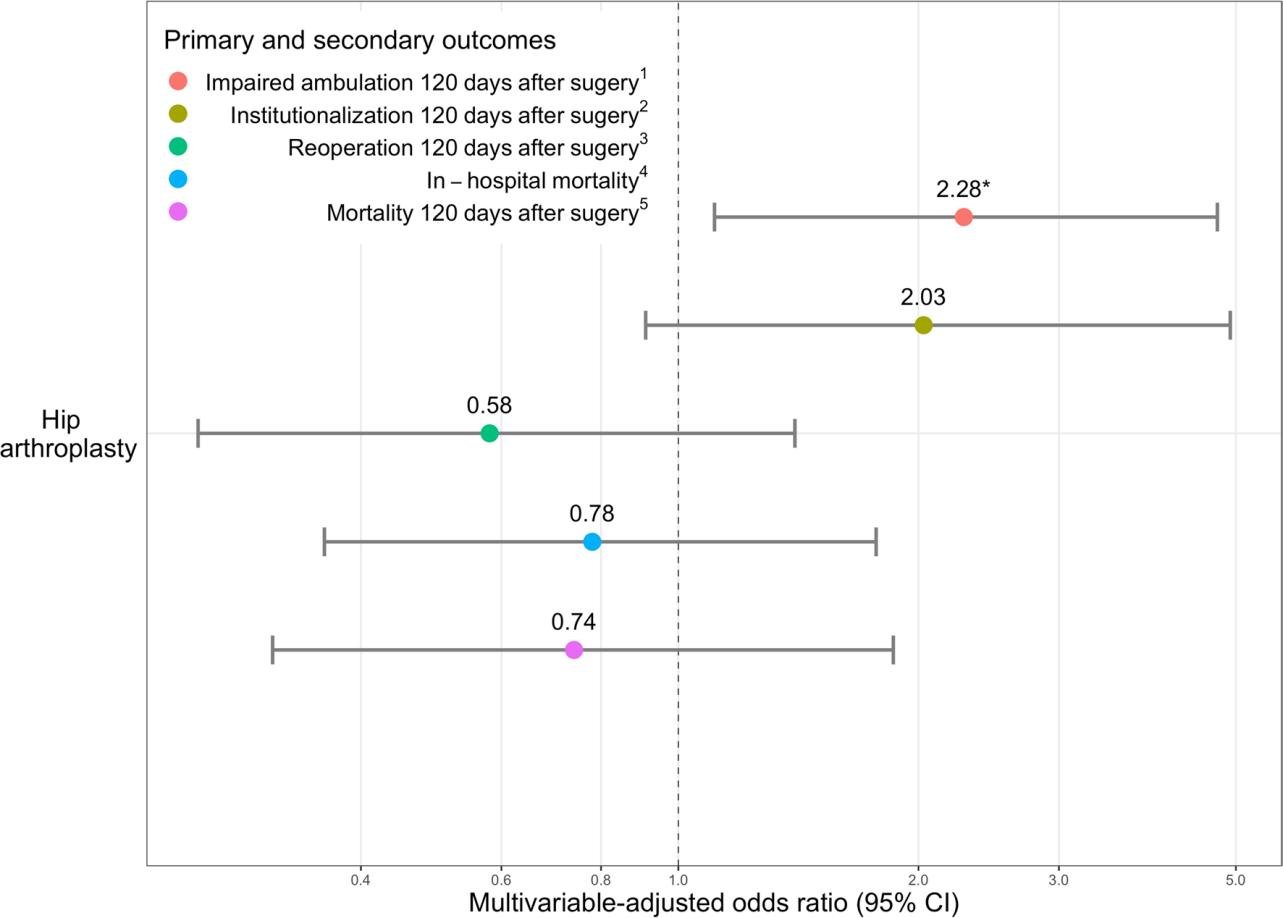
Multivariable analyses with adjusted odds ratios and 95% confidence intervals (CI) for the surgical treatment of hip arthroplasty as predictors of in-hospital mortality as well as impaired ambulation, institutionalization, reoperation, and mortality at follow-up. The model was adjusted for sex, age, prefracture residential and mobility status, ISAR score, use of prefracture anticoagulation medication, and ASA class. ASA class, American Society of Anesthesiologists classification; ISAR, Identification of Seniors at Risk. *p < 0.05. Data availability: ^1^*n* = 375, ^2^*n* = 351, ^3^*n* = 484, ^4^*n* = 990, ^5^*n* = 378

The prognostic value of surgical treatment (IF or HA) for primary and secondary outcomes adjusted for patient demographics and baseline characteristics was analyzed in multiple individual linear or logistic regression analyses. After adjusting for key covariates, including prefracture ambulatory capacity, HA was a significant independent predictor of reduced mobility at the 120-day follow-up point (OR 2.28, 95% CI 1.11–4.74). Thus, the mobility of patients treated with HA at follow-up was 2.28 times more often impaired than that of those who received IF. Furthermore, no independent association of surgical treatment with institutionalized living status, reoperation rate, or mortality was observed (Fig. 2). Additionally, no independent associations of surgical treatment with the EQ-5D Index Score at day seven during in-hospital care (data available for 827 patients) or at 120 days after index surgery (data available for 296 patients) were found (ß = 0.010, 95% CI − 0.030–0.050, *p* = 0.615 and ß = 0.004, 95% CI − 0.057–0.064, *p* = 0.903, respectively).

## Discussion

Nondisplaced FNFs commonly occur in geriatric patients, and their incidence will further increase due to demographic changes in industrialized countries. However, only 60% of patients recover to their prefracture walking abilities within the first six months after surgery [25]. To contribute to an ongoing international debate regarding the preferred surgical treatment of geriatric nondisplaced FNFs [26], we analyzed data from 1,727 patients from the ATR-DGU who received either IF or HA.

The particularly high number of patients with impaired ambulation before fracture (IF Group 43.0%, HA Group 34.4%) and the further postoperative decrease at short-term follow-up (IF Group 19.6%, HA Group 10.5%) may be related to the strict definition of ambulation without impairment (i.e., ability to walk without any assistive devices). However, a similar decrease of 59% to 26% in the proportion of individuals able to walk without aids at the one-year follow-up after hip fracture has been observed in another study [27]. Moreover, at the time of follow-up, HA was a significant predictor of impaired mobility (OR 2.28); notably, the model controlled for prefracture ambulatory capacity. This observation contrasts with other studies reporting either no differences between screw osteosynthesis and hemiarthroplasty or superior functional outcome for hemiarthroplasty at short-term follow-up [14, 28]. The specific type of osteosynthesis might have particularly contributed to the superior mobility outcome in the present study. Almost 60% of patients in the IF group received dynamic hip screws (DHSs), for which superior functional outcomes compared to those associated with screw osteosynthesis have been reported [29]. We acknowledge that the assessment of mobility within a short follow-up period of 120 days may have a limited capacity to predict long-term functional outcomes, which is particularly relevant during the process of establishing treatment recommendations. However, the functional outcomes, including mobility rates, of patients treated with screw osteosynthesis either remained similar or improved compared to those of patients treated with hemiarthroplasty within two to three years after surgery in other studies [14, 28]. Thus, we infer that favorable short-term results may well translate into successful long-term functional outcomes of IF, particularly when DHSs or comparable implants are used.

Furthermore, we observed a higher unadjusted institutionalization rate in the HA group at the 120-day postoperative follow-up point. Previous studies identified older age, poorer general health and impaired prefracture functional status as determinants of discharge to residential/nursing care and long-term need for supported living arrangements following hip fractures [30–32]. In line with these findings, the adjusted multivariable analysis did not show an independent association of surgical treatment with institutionalization rate. Therefore, we believe the observed higher institutionalization rate may be due to the older age, poorer general condition (higher ISAR score) and more impaired prefracture ambulatory capacity of patients in the HA group rather than the surgical treatment itself. In addition, patients undergoing HA might have been required to improve their underlying health conditions more frequently before receiving clearance to receive anesthesia, which, in turn, might have prolonged the time to surgery and, thereby, increased the LOS in this group [33, 34]. Osteosynthetic treatment of FNFs is less invasive; therefore, patients in the IF group with better general health might have received preoperative clearance for anesthesia earlier than those in the HA group [35]. However, to conclusively elucidate the independent influence of surgical treatment on residential status and LOS, prospective trials are needed.

Our unadjusted and adjusted reoperation rates showed no differences between the IF and HA treatments. In a recently published randomized controlled trial (RCT) of impacted FNFs with a 36-month follow-up, Wei et al. [14] also observed no significant differences in the reoperation rate between the IF and hemiarthroplasty groups. These findings, however, are not in accordance with a Norwegian study [28] that reported a reoperation rate of 20% after screw osteosynthesis versus 5% after hemiarthroplasty of nondisplaced FNFs (*p* = 0.002). In that study, the standard treatment of nondisplaced FNFs was osteosynthesis with two cancellous screws [28], while in the present cohort, a larger proportion of patients in the IF group did not undergo treatment with screw osteosynthesis. The greater biomechanical stability of DHSs compared to cancellous screws may reduce reoperation rates and result in a higher overall success rate [36]. Notably, a subgroup analysis of the FAITH trial showed that 13.2% of nondisplaced FNF patients who underwent osteosynthetic treatment (49.9% cancellous screws, 50.1% DHS) required HA in the 24 months following the initial procedure [37]. Although no significant differences were found, we also observed that secondary THR or hemiarthroplasty were the major causes of reoperation in the IF group. However, a subgroup analysis of the nondisplaced FNF patients with IF conducted to elaborate the results of the different osteosynthetic treatments (e.g., cancellous screws versus DHS) was not performed in the FAITH trial [37] or in our study. The two large RCTs currently being conducted in Sweden (HipSTHeR trial [38]) and in Denmark (SENSE trial [39]) comparing IF including screw fixation or DHS with THR or hemiarthroplasty might achieve the required sample size to allow such subgroup analyses.

Furthermore, overall satisfactory long-term HRQoL has been described in the literature, especially for older patients treated for nondisplaced FNFs with IF as well as for those undergoing primary elective THR [14, 40]. In line with recent findings [14, 28], we observed that the difference in HRQoL between the IF and HA groups remained proportionate throughout the follow-up period and that the EQ-5D index scores did not differ between the groups.

In addition to no differences in HRQoL, in line with previous studies [28, 41], we also found similar short-term mortality in both treatment groups. The short-term follow-up mortality rates observed in the present study (IF 7.5%, HA 9.7%) were slightly higher than the four-month mortality rates of 6.6% observed in patients with displaced FNFs treated with osteosynthesis and 7.1% in those treated with hemiarthroplasty reported by Gjertsen et al. [41]. However, the ASA physical status in the current study indicated inferior overall preoperative health compared to that among the individuals included in the study conducted by Gjertsen et al. [41], which might account for the minor differences in early mortality between the studies. Notably, while surgical treatment was not an independent predictor of mortality in the current study, Gjertsen et al. [41] observed lower HRQoL at a four-month follow-up in patients with displaced FNFs treated with IF compared to the HRQoL of those treated with hemiarthroplasty. Thus, we believe that when the eligibility criteria for IF are chosen appropriately, comprising the exclusion of displaced FNFs, osteosynthetic treatment, especially if an alternative method to screw fixation is used, might represent an adequate treatment option for elderly FNF patients. In addition, avoiding screw osteosynthesis may be associated with favorable functional outcomes and fewer reoperations; however, RCTs with an additional focus on patient-reported outcome measures (PROMs) [38, 39] are required to compare different types of osteosynthesis for geriatric nondisplaced FNFs before final treatment recommendations can be made.

We note several limitations mainly attributed to the retrospective study design, although data collection for the ATR-DGU was prospective. Preoperative patient mobility was not objectively measured. Furthermore, PROM data (EQ-5D questionnaire) were available by day seven after hospital admission. This collection of subjective data in combination with the absence of repeated measurements preintervention and postintervention might have resulted in retrospective reporting bias [42]. However, we controlled for potential confounders, which improves the generalizability of the results obtained in observational studies [43]. Nonetheless, we were unable to analyze the effect of potentially influential factors that were not recorded in the initial ATR-DGU, such as posterior tilt on preoperative radiographic imaging, which is associated with fixation failure in particular [37]. The most common complications after IF of nondisplaced FNFs are nonunion and AVN, which often develop two to three years after treatment [44]. Therefore, we might have missed associated reoperations due to our follow-up period of four months. Nonetheless, patients’ functional capabilities upon hospital discharge have proven to be a strong predictor of long-term functional status [45]. Missing patient data at follow-up (e.g., due to transfer to nursing facility), as observed in the current study, are common in geriatric hip fracture studies, and similar response rates have been reported in a four-month follow-up period [41]. Furthermore, a postoperative 120-day follow-up rate of only 48% in the ATR-DGU has been described and attributed to the voluntary nature of the respective data acquisition [46]. Therefore, selection bias cannot be excluded.

## Conclusion

After a short-term follow-up, patients who received IF following nondisplaced FNFs had superior mobility and similar reoperation rates, EQ-5D index scores, and mortality along with shorter in-hospital care durations than those who received HA. However, RCTs with a longer follow-up period are required to confirm superior mobility rates for IF in the long term and to provide objective evidence necessary to establish treatment guidelines for nondisplaced FNFs in geriatric patients.

## Supplementary Information

Below is the link to the electronic supplementary material.

Supplementary file1 (DOCX 14 KB)

## Data Availability

The data used to support the findings of this study are provided by the ATR-DGU and maintained by the AUC. Data are available from the AUC for researchers who meet the criteria for access to confidential data. Requests for access to these data should be made to the AUC.
