# Supplementary material for: Internal fixation versus hip arthroplasty in patients with nondisplaced femoral neck fractures: short-term results from a geriatric trauma registry
Source: Eur J Trauma Emerg Surg. 2021 Oct 5;48(3):1851–9. doi: 10.1007/s00068-021-01801-1 (PMC9192444; doi:10.1007/s00068-021-01801-1)
Supplement: Supplementary file 1 — Supplementary file1 (DOCX 14 KB) [file 68_2021_1801_MOESM1_ESM.docx]

# Supplementary material

**Supplement 1.** Types of reoperation during in-hospital care and 120-day follow-up.

|  | **Internal fixation**  **(n = 449)** | **Hip arthroplasty**  **(n = 1278)** |
| --- | --- | --- |
| **Reoperation during in-hospital care †** | | |
| Soft tissue debridement and lavage | 1 | 19 |
| Reduction | 0 | 3 |
| Revision | 4 | 3 |
| Secondary hemiarthroplasty | 3 | 0 |
| Peri-implant/peri-prosthetic fracture | 1 | 2 |
| Others (local) | 3 | 13 |
| **Total** | 12 | 40 |
| **Reoperation during 120-day follow-up ‡** | | |
| Soft tissue debridement and lavage | 0 | 10 |
| Reduction | 0 | 3 |
| Revision | 5 | 4 |
| Secondary hemiarthroplasty | 6 | 0 |
| Secondary THR | 3 | 0 |
| Removal hemiarthroplasty and secondary THR | 0 | 1 |
| Removal THR and secondary hemiarthroplasty | 0 | 1 |
| Peri-implant/peri-prosthetic fracture | 1 | 4 |
| Others (local) | 2 | 7 |
| **Total** | 17 | 30 |

THR, total hip replacement. The numbers of conducted reoperations are displayed herein when multiple reoperations per patient were conducted. † Data missing for two patients in the internal fixation group and three patients in the hip arthroplasty group. ‡ Data missing for 210 patients in the internal fixation group and 672 patients in the hip arthroplasty group.
